# Prognostic and Predictive Values of Mismatch Repair Deficiency in Non-Metastatic Colorectal Cancer

**DOI:** 10.3390/cancers13020300

**Published:** 2021-01-15

**Authors:** Zhaohui Jin, Frank A. Sinicrope

**Affiliations:** Division of Oncology, Mayo Clinic and Mayo Comprehensive Cancer Center, Rochester, MN 55905, USA; jin.zhaohui@mayo.edu

**Keywords:** mismatch repair deficiency, microsatellite instability, non-metastatic colorectal cancer, prognostic, predictive

## Abstract

**Simple Summary:**

A subset of colorectal cancers (CRCs) displays deficient DNA mismatch repair (dMMR) that leads to microsatellite instability (MSI). These tumors have distinct clinicopathological features and have been associated with a more favorable prognosis. Knowledge of mismatch repair (MMR) status has important implications for disease diagnosis, surgical intervention, and adjuvant treatment decisions.

**Abstract:**

Colorectal cancer (CRC) is the third most commonly diagnosed cancer worldwide. Universal MMR/MSI testing is standard of care for all patients with newly diagnosed CRC based on multi-society guidelines in the United States. Such testing is intended to identify patients with Lynch Syndrome due to a germline mutation in an MMR gene, but also detects those with sporadic dMMR/MSI-high CRCs. The prognostic utility of MMR/MSI status in non-metastatic colorectal cancer has been studied extensively, yet more limited data are available for its predictive utility. Results have not been entirely consistent due to potential stage-related differences and limited numbers of dMMR/MSI-H patients included in the studies. In this review, we summarize the current evidence for the prognostic and predictive value of dMMR/MSI-H in non-metastatic CRC, and discuss the use of this biomarker for patient management and treatment decisions in clinical practice.

## 1. Introduction

Colorectal cancer (CRC) is the second most common cancer diagnosed in women and the third in men worldwide, with more than 1.8 million new cases and approximately 900,000 deaths in 2017 [1]. It is estimated that 147,950 new CRC cases will be diagnosed in the United States with 53,200 deaths in 2020 [2]. Approximately 15% of CRCs show deficient DNA mismatch repair (dMMR) that results in microsatellite instability (MSI). These tumors are frequently poorly differentiated with mucinous features or a medullary growth pattern, although they can also resemble more typical CRCs. CRCs with dMMR have earlier stage at diagnosis compared to proficient MMR (pMMR) tumors [3,4]. Due to the loss of mismatch repair (MMR) function, these tumors accumulate a high mutational burden with abundant mutation-derived neoantigens that attract tumor infiltrating lymphocytes (TILs) [5,6]. Non-metastatic CRCs with dMMR/MSI generally have better stage-adjusted prognosis compared to pMMR tumors, and data suggest that MMR status may also be predictive of tumor responsiveness to different treatments [7,8].

Here, we review the role of dMMR/MSI in CRC development, and the prognostic and predictive value of dMMR/MSI in non-metastatic CRCs.

## 2. The Role of dMMR/MSI in CRC Development

The first multi-step model of CRC carcinogenesis considered adenomatous polyposis coli (*APC*) gene inactivation as the initial step followed by *KRAS* gene mutation and chromosome 18q loss of heterozygosity that further promotes the growth of precancerous adenomas. *P53* gene inactivation mediates the adenoma-to-carcinoma transition. However, an important alternative pathway was identified where tumors showed microsatellite instability (MSI) due to deficient DNA mismatch repair (dMMR).

MSI is characterized by short sequence repeats (SSRs) or short tandem repeats (STRs) of repeated DNA sequences with various lengths [9]. Microsatellites are widely distributed throughout the genome in a non-random fashion and are prone to mutations during DNA replication [10,11]. In 1993, an analysis of 130 matched CRC tumors and adjacent normal tissues identified differences in polymerase chain reaction (PCR) products whereby 12% of tumors had bands that were shorter in length (band-shift) [12]. Sequencing of these bands by the Perucho lab revealed that they contained simple repetitive sequences termed microsatellites. Further study demonstrated that tumors with this type of mutation had unique characteristics that led to the hypothesis that these tumors could be hereditary. Simultaneously, Thibodeau et al. examined somatic instability in CRCs in human chromosomes 5q, 15q, 17p, and 18q and identified differences between normal tissue and tumor DNA that linked MSI directly with CRC carcinogenesis [13]. MSI was found in association with CRCs arising in hereditary non-polyposis colorectal cancer (HNPCC) including other cancer types, suggesting that cancers developing in HNPCC have a common pathogenesis via MSI [14]. Almost at the same time, Aaltonen’s lab confirmed widespread MSI in familial CRC; however, MSI was also identified in 13% of sporadic CRC cases [15]. This suggested that MSI is a pathogenic pathway shared by both hereditary and sporadic CRC. MSI CRCs are characterized by a large number of mutations at microsatellite sequences, and are commonly located in the proximal colon, have poorly differentiated histology with mucinous features, and appear to have better clinical outcomes [14,16].

The mismatch repair (MMR) system consists of a family of enzymes that detect DNA replication errors (such as mismatches between the two strands of DNA). The MMR system includes *MHL1*, *MSH2*, *MSH6*, and *PMS2* genes. Approximately 90% of germline mutations are detected in *MLH1* and *MSH2* genes. Germline mutations in *MLH1* were first identified in multiple familial CRC kindreds [17,18]. Human *PMS1* and *PMS2* genes were subsequently implicated in familial CRC, although the role of *PMS1* in CRC remains unclear [19]. It took longer to confirm the role of *MSH6* in MSI CRC due to delayed onset of cancer that obscured the initial effort of discovery [20]. The *EpCAM* (Epithelial cellular adhesion molecule) gene is located upstream of *MSH2,* and germline 3′ end deletion of the *EpCAM* gene leads to hypermethylation of the *MSH2* promoter (constitutional epimutation) and MSI [21]. Germline mutations of these genes (*MLH1*, *MSH2*, *MSH6*, *PMS2*, and *EpCAM*) lead to HNPCC, also known as Lynch syndrome.

Although progress in recognizing the role of MMR deficiency in MSI CRC is mainly based on the studies in familial CRC (HNPCC) population, these patients represent only 3% of all CRCs [22]. Approximately 12–17% of all CRCs have MSI which indicates the majority of MSI CRCs are sporadic [7,23]. The sporadic MSI CRCs have unique characteristics including later onset of cancer without familial clustering, frequent *BRAF*^V600E^ mutations, and better clinical outcomes as also found for familial MSI tumors [24,25]. Most sporadic MSI CRCs show loss of MLH1 and PMS2 proteins and the mechanism of MSI in these tumors is due to hypermethylation of the *MLH1* gene promoter typically in association with the CpG island methylator phenotype (CIMP) [26]. Approximately 50% of human genes have promotor regions embedded in the clusters of cytosine-guanosine residues called CpG islands, and cytosines in the CpG island can be methylated, thus leading to gene silencing [27]. *BRAF*^V600E^ occurs exclusively in sporadic dMMR/MSI CRCs in association with hypermethylation of the *MLH1* gene promoter, often with the CpG island methylator phenotype (CIMP) [28,29]. However, 2.5–3.9% of patients with MSI CRCs do not have germline mutation or *MLH1* methylation and these tumors have been found to have double somatic MMR mutations [22,30,31,32]. These double somatic MSI CRCs have a higher frequency of *PIK3CA* somatic mutation [33].

Genes containing microsatellites are prone to mutations due to dMMR. Such genes include those regulating cell proliferation (*TGFβR2*, *GRB1*, *TCF-4*, *WISP3*, *IGFIIR*), cell cycle or apoptosis (*caspase-5*, *BAX*, *FAX*, *PTEN*), and DNA repair (*CHK1*, *MLH3*, *RAS50*, *MSH3*). Mutations in these genes predispose to CRC development [34].

dMMR/MSI CRCs typically harbor increased numbers of both intraepithelial and peritumoral lymphocytes that represent a response to neoantigens generated by the high mutational burden secondary to MSI [6,35,36]. HNPCC-associated CRCs are more commonly seen in men and usually develop at an earlier age than sporadic dMMR/MSI CRCs (average age of cancer onset, 52.9 vs. 70.8 years) [37,38]. HNPCC patients have high risk for synchronous and metachronous CRCs. One study reported that approximately 7% of patients with HNPCC had synchronous CRCs at the time of diagnosis [39]. For HNPCC patients who had a segmental resection of the first colon cancer, 62% developed a metachronous CRC within 30 years of follow-up, suggesting the need of prophylactic total colectomy in this population [40].

It is critical to identify dMMR/MSI CRCs and currently there are different approaches for detection, including immunohistochemistry (IHC), polymerase chain reaction (PCR)-based methods, and next generation sequencing (NGS). IHC directly evaluates the MMR protein presence/absence in the tumor cells while PCR-based tests use a set of primers (most commonly including two mononucleotide probes (BAT25 and BAT26) and three dinucleotide probes (D2S123, D5D346, D17S250)) to check for PCR products size differences between normal and tumor tissues (band-shift). These two approaches are sensitive and specific with high concordance rate (92–97%) [41,42]. Importantly, there is a small percentage of dMMR CRCs that show intact expression of MMR proteins at IHC, yet have a dysfunctional MMR protein that is due to a missense mutation in an MMR gene. To detect such cases, some experts recommend both IHC and PCR-based tests for dMMR/MSI screening [43,44]. More recently, massive parallel NGS demonstrated the capability of accurately detecting MSI. NGS is able to detect MSI simultaneously in a large number of microsatellite loci. One study evaluated 11,573 solid tumor specimens with NGS and demonstrated a high concordance rate with PCR and IHC results (97%) [45]. Of note, this approach requires specific algorithms and computational methods which can vary with different NGS platforms, although high sensitivity and specificity were confirmed [46,47]. As NGS-guided precision oncology is becoming part of routine clinical practice, this approach is frequently utilized to identify MSI cancers. Liquid biopsy has emerged as a comprehensive approach to characterize the molecular features of tumors by testing cell-free DNA (cfDNA, i.e., fragments of DNA that are shed into the bloodstream from dividing cells during cell proliferation or cell death). One study showed that MSI testing using cfDNA has an overall accuracy of 98.4% [48]. This method is currently incorporated into clinical practice, especially for those patients who have insufficient tumor tissue for IHC, PCR, or NGS tests.

## 3. Prognostic Value of dMMR/MSI in Non-Metastatic Colorectal Cancer

CRCs with dMMR/MSI are more commonly seen in early stage disease and the incidence is reported to be 20% in stage II, 11% in stage III, and 3.5% in metastatic disease, suggesting that MSI CRCs have a reduced tendency for distant metastasis [49]. Substantial evidence supports that dMMR/MSI is a strong prognostic marker in early stage CRCs with a favorable impact on survival. The quick and simple and reliable (QUASAR) phase III clinical trial evaluated the role of fluorouracil-based adjuvant chemotherapy in stage II colon cancer. In a subsequent analysis, there were 1913 stage II and III CRCs patients, of which 218 (11.4%) patients were found to have MSI tumors and only 10 were stage III patients. The proportion of MSI tumors varied significantly by primary site: 179 of 695 (26%) right-sided colon, 22 of 685 (3%) left-sided colon, and 3 of 407 (1%) rectal cancers. In a subgroup analysis in stage II patients, dMMR/MSI was associated with a significantly decreased risk of tumor recurrence (risk ratio (RR) 0.53, 95% confidence interval (CI): 0.29–0.67, *p* < 0.001) [50]. In a pooled analysis of 1027 stage II and III colon cancer patients of which 165 (16.1%) showed dMMR/MSI, the presence of dMMR/MSI was associated with significantly improved disease-free survival (DFS) (hazard ratio (HR) 0.51, 95% CI: 0.29–0.89, *p* = 0.009) and overall survival (OS) (HR 0.47, 95% CI: 0.26–0.83, *p* = 0.004) in patients who did not receive adjuvant chemotherapy [51]. In the Adjuvant Colon Cancer Endpoint (ACCENT) database analysis that included 17 adjuvant clinical trials, 524 of 2270 (23.1%) stage II colon cancer patients were identified to have dMMR/MSI tumors. dMMR/MSI was associated with improved overall survival (OS) (HR 0.27, *p* = 0.01) and time to recurrence (TTR) (HR 0.27, *p* = 0.01) in stage II colon cancer patients following surgical resection compared to patients with MMR proficient (pMMR)/microsatellite stable (MSS) disease [52].

The prognostic value of dMMR/MSI in stage III CRC is less clear compared to stage II and conflicting data exist. In a pooled analysis of 2141 stage II (*n* = 778) and stage III (*n* = 1363) colon cancer patients, 344 (16.1%) had dMMR/MSI tumors that were associated with delayed TTR (HR 0.72, 95% CI: 0.56–0.91, *p* = 0.005) and improved disease-free survival (DFS) (HR 0.80, 95% CI: 0.64–0.99, *p* = 0.035) and OS (HR 0.79, 95% CI: 0.64–0.99, *p* = 0.031) vs. pMMR/MSS tumors. Further subgroup analysis showed that the prognostic benefit only achieved statistical significance in stage III cancers for TTR (HR 0.72, 95% CI: 0.54–0.97, *p* = 0.024) [53]. In the PETACC3 adjuvant study with 1404 stage II and III colon cancer patients, 15% of these patients had dMMR/MSI tumors with 22% being stage II and 12% stage III cancers. Patients with dMMR/MSI cancers had better relapse-free survival (RFS) (HR 0.54, 95% CI: 0.37–0.81, *p* = 0.003) and OS (HR 0.43, 95% CI: 0.27–0.70, *p* = 0.001) compared to MSS CRCs by multivariable analysis [54]. In a pooled analysis of stage II and III colon cancer patients from the National Surgical Adjuvant Breast and Bowel Project (NSABP) clinical trials C-07 (*n* = 1836) and C-08 (*n* = 463), dMMR/MSI tumors were associated with better RFS (HR 0.48, 95% CI: 0.33–0.70, *p* = 0.0001) and OS (HR 0.64, 95% CI: 0.46–0.89, *p* = 0.0084) [55]. Furthermore, dMMR/MSI in stage III cancers was also found to be associated with better survival after recurrence (SAR). A pooled analysis of seven phase III clinical trials included 2630 patients who had resected stage III colon cancer with subsequent tumor recurrence. Among these, 271 patients (10.3%) had dMMR/MSI tumors which were associated with better SAR (HR 0.82, 95% CI: 0.69–0.98, *p* = 0.029) compared to MSS tumors. Of note, *BRAF*^V600E^ was associated with poorer prognosis in both dMMR/MSI and in pMMR/MSS stage III colon cancers [56].

Other studies found that dMMR/MSI was not prognostic in stage III colon cancer. The prognostic impact of dMMR/MSI was studied in 2580 patients with stage III colon cancer who participated in the phase III adjuvant trial of FOLFOX-based chemotherapy (North Central Cancer Treatment Group (NCCTG) N0147). Among 2580 participants, 314 (12%) patients had dMMR/MSI tumors which made this the largest dMMR/MSI stage III CRC cohort reported to date. This study revealed that dMMR/MSI was not associated with better DFS compared to pMMR/MSS patients which did not change after adjustment for clinical variables, *BRAF* or *KRAS* status (HR 0.82, 95% CI: 0.64–1.07, *p* = 0.14). However, a statistically significant interaction was found between MMR status and disease-free survival (DFS) by primary tumor sidedness. Significantly better DFS was seen in dMMR tumors of the proximal colon (HR 0.71, 95% CI: 0.53–0.94, *p* = 0.018) but not in the distal colon (HR 1.71, 95% CI: 0.99–2.95, *p* = 0.056), and these results were validated in an independent cohort (CALGB 89803) [57]. Subsequent analysis of the PETACC3 study revealed that dMMR/MSI status was associated with better RFS (HR 0.48, 95% CI: 0.34–0.69, *p* < 0.001) and OS (HR 0.47, 95% CI: 0.31–0.72, *p* < 0.001) in the overall study population. However, the prognostic effect was mainly driven by the benefits seen in stage II disease since only a borderline benefit was seen for RFS in stage III patients [58].

Studies also suggested that other prognostic markers such as node stage (N2 versus N1), and *RAS* and *BRAF* mutation status may also contribute to prognosis in dMMR/MSI CRC. One study showed that N2 disease (≥4 positive lymph nodes) among dMMR/MSI stage III CRCs was associated with worse clinical outcomes [57]. Due to the relatively small numbers of dMMR/MSI CRCs included in individual studies, inconsistent results have been reported. In the ACCENT database of 17 adjuvant chemotherapy trials, *BRAF*^V600E^ was associated with worse SAR (HR 2.65, 95% CI 1.67–4.21, *p* < 0.0001) in dMMR/MSI CRCs. Although *KRAS* mutations and *BRAF*^V600E^ mutation were associated with worse DFS in the NCCTG N0147 study, their prognostic value was limited to pMMR/MSS tumors [57]. This result was confirmed in a pooled analysis of 4411 stage III colon cancer patients from the NCCTG N0147 and PETACC8 studies with 477 dMMR/MSI tumors [59].

Recently, a systematic review and meta-analysis included 51 studies with 28,331 stage II and III CRC patients. The 16.4% of patients found to have dMMR/MSI CRCs had improved DFS (HR 0.67, 95% CI: 0.59–0.75, *p* < 0.001) and OS (HR 0.74, 95% CI: 0.68–0.82, *p* < 0.001) and importantly, the observed DFS and OS benefits were similar in both stage II and stage III disease [60]. However, another meta-analysis was performed that included only stage III CRCs from 36 studies consisting of both randomized clinical trials (RCT) and non-RCTs. This study found that dMMR/MSI had no prognostic impact for OS, DFS, and disease specific survival (DSS) [61]. The discrepancies among these studies are likely multifactorial and include data from non-randomized and non-study cohorts, different adjuvant chemotherapy regimens, small numbers of dMMR/MSI CRC patients enrolled in each study, and other factors contributing to heterogeneity of the patient populations. One observation appears consistent, which is that the prognostic impact of dMMR/MSI declines with regional and distant metastatic disease such that a favorable prognosis exists in stage II CRC while the effects diminish in stage III disease.

Why does the prognostic value of dMMR/MSI decrease in stage III CRC? It is believed that the prognostic benefits from dMMR/MSI rely on the immunological reaction associated with dMMR/MSI tumors. Enhanced lymphocytic infiltration with an immunoreaction is detected in dMMR/MSI CRCs and this leads to increased host anti-tumor immunity to suppress tumor metastasis [62]. The observed decreased incidence of dMMR/MSI CRCs with advancing disease stage is consistent with this hypothesis. It is speculated that with disease progression and development of metastasis, mechanisms of immune evasion develop that enable dMMR/MSI tumors to evade immune surveillance with loss of a prognostic advantage. This is seen in stage IV CRCs with dMMR/MSI where no prognostic advantage was found [63]. Table 1 lists the recent studies evaluating prognostic value of dMMR/MSI in CRC.

## 4. Predictive Value of dMMR/MSI for Adjuvant Chemotherapy in Non-Metastatic Colorectal Cancer

Initial retrospective small studies suggested that fluorouracil (5-FU)-based adjuvant chemotherapy was beneficial for stage II and III CRC cancer irrespective of the MMR status [64,65,66]. A retrospective study including 891 consecutive stage III CRC with median follow-up of 54 months suggested that adjuvant chemotherapy significantly improved survival in patients with dMMR/MSI cancers [67]. This study only used one microsatellite marker to identify MSI disease and there were only 63 dMMR/MSI patients included in the study.

Ribic et al. studied specimens from prospective, randomized trials of 5-FU-based adjuvant chemotherapy to further evaluate the predictive utility of dMMR/MSI [68]. The study included 570 patients with 95 (16.7%) dMMR/MSI stage II and III colon cancer patients, and 287 patients (42 dMMR/MSI) who did not receive adjuvant treatment. MSI status was determined by a PCR-based assay with multiple probes. dMMR/MSI was associated with a better 5-year survival rate among patients who did not receive adjuvant chemotherapy (HR 0.31, 95% CI: 0.14–0.72, *p* = 0.004). However, 5-FU-based adjuvant chemotherapy did not improve 5-year OS (HR 1.07, 95% CI: 0.62–1.86, *p* = 0.80) in patients with dMMR/MSI tumors while it seemed to benefit those with pMMR/MSS tumors (HR 0.72, 95% CI 0.53–0.99, *p* = 0.04). The lack of benefit seemed to be similar in both stage II and stage III dMMR/MSI cancers in a subgroup analysis.

Another pooled analysis combined five randomized adjuvant clinical trials with 457 stage II and III colon cancer patients and confirmed the lack of benefit of 5-FU as adjuvant therapy in dMMR/MSI tumors [51]. Seventy dMMR/MSI patients were included in the study and 5-FU-based adjuvant treatment failed to improve DFS (HR 1.39, 95% CI: 0.46–4.15, *p* = 0.56) although it significantly improved DFS for pMMR/MSS patients (HR 0.67, 95% CI: 0.48–0.93, *p* = 0.02). To further evaluate the stage-specific predictive utility of dMMR/MSI, the study combined these patients with the previously reported dataset [68] that resulted in 1027 stage II and III patients with 165 dMMR/MSI cases. Although adjuvant chemotherapy showed a DFS benefit in stage III pMMR/MSS patients (HR 0.64, 95% CI: 0.48–0.84, *p* = 0.001), it did not lead to a DFS benefit in either stage II (HR 2.30, 95% CI 0.29–0.89, *p* = 0.09) or stage III (HR 1.01, 95% CI 0.26–0.83, *p* = 0.98) dMMR/MSI colon cancers. Interestingly, there was a detrimental effect on OS observed from receipt of adjuvant chemotherapy in stage II dMMR/MSI colon cancer patients (HR 2.95, 95% CI: 1.02–8.94, *p* = 0.04). A Korean study including 860 stage II colon cancer patients with 126 (14.7%) dMMR/MSI cases also confirmed that adjuvant chemotherapy did not improve DFS (HR 0.557, *p* = 0.254) in this patient population, although OS seemed to be improved (HR 0.288, *p* = 0.033) [69].

Analysis of 1913 stage II CRCs from the QUASAR study including 218 dMMR/MSI cases, showed that MMR status is prognostic but is not predictive for the outcome of adjuvant chemotherapy (odds ratio (OR) 0.81, 95% CI: 0.29–2.22) [50]. However, this study had a very small number of events (only 15) which makes it difficult to interpret the findings. Another study pooled data from two adjuvant trials (CALGB 9581 and 89803) that included 1852 patients of which 330 were dMMR/MSI and included 199 (21.1%) stage II and 131 (14.3%) stage III CRC patients. MMR status was found to be prognostic but not predictive of outcome of adjuvant treatment consisting of infusional 5-FU with an irinotecan-based regimen) [70].

Another study evaluated the predictive utility of dMMR/MSI in 2141 stage II and III colon cancer patients of which 344 were dMMR/MSI and were treated with 5-FU-based adjuvant chemotherapy [53]. This study found that 5-FU-based adjuvant treatment was associated with a reduced 5-year recurrence rate (22% versus 37%, *p* = 0.044) especially at distant sites (11% versus 29%, *p* = 0.011), including the liver (22% versus 56%, *p* = 0.005). The benefit of adjuvant chemotherapy was limited to stage III disease. A subgroup analysis suggested that the benefit of 5-FU-based adjuvant treatment among dMMR/MSI stage III tumors was limited to patients with suspected hereditary, but not sporadic dMMR/MSI CRCs.

Adjuvant treatment for CRC evolved and fluorouracil, leucovorin, and oxaliplatin (FOLFOX) became the standard of care after the landmark MOSAIC study (multicenter international study of oxaliplatin/5-fluorouracil/Leucovorin (LV) in the adjuvant treatment of colon cancer) which showed benefits in DFS and OS for the addition of oxaliplatin to the 5-FU/leucovorin regimen [71].

A small retrospective study of 233 stage III colon cancer patients included 32 dMMR/MSI cases. Patients had either 5-FU/LV (*n* = 20) or FOLFOX (*n* = 12) as adjuvant treatment [72]. The addition of oxaliplatin was associated with improved DFS in dMMR/MSI patients compared to 5-FU/LV only treatment (HR 0.17, 95% CI: 0.04–0.68, *p* = 0.01). In an update of the MOSAIC study with 9.5 years median follow-up, 95 dMMR/MSI stage II/III CRC cases were identified among 1008 patients. FOLFOX as adjuvant treatment was associated with a trend toward improved DFS (HR 0.48, 95% CI: 0.21–1.12, *p* = 0.088) and OS (HR 0.41, 95% CI: 0.16–1.07, *p* = 0.069) among patients with dMMR/MSI tumors [73]. A retrospective study known as AGEO included 433 dMMR/MSI stage II and III colon cancer patients and evaluated the impact of adjuvant 5-FU or FOLFOX treatment [74]. In the study population, oxaliplatin-based adjuvant chemotherapy was associated with a trend toward improved DFS (HR 0.13 95% CI 0.02–1.05, *p* = 0.06) while 5-FU/LV alone did not show a DFS benefit. A subgroup analysis showed that the DFS benefit from oxaliplatin-based adjuvant treatment was limited to stage III colon cancers (HR 0.41, 95% CI: 0.19–0.87, *p* = 0.02). Most recently, a pooled analysis of C-07 and MOSAIC trial including 1625 stage III colon cancer patients with 185 dMMR/MSI cases revealed that the addition of oxaliplatin to fluoropyrimidine adjuvant treatment significantly improved OS (HR 0.52, 95% CI: 0.28–0.93) and DFS (HR 0.47, 95% CI: 0.27–0.82) compared to fluoropyrimidine alone treatment. Interestingly, the survival benefit from oxaliplatin based adjuvant treatment seemed to be more prominent in dMMR/MSI patients [75]. These data suggested dMMR/MSI stage III CRC patients benefit from oxaliplatin-based adjuvant treatment.

Given the favorable prognosis of dMMR/MSI in stage II CRC and lack of clear evidence of a survival benefit from 5-FU-based adjuvant treatment, the current guidelines do not recommend adjuvant treatment for stage II dMMR/MSI colon cancer. For stage III CRC with dMMR/MSI, oxaliplatin-based adjuvant treatment is considered standard of care due to the diminished prognostic benefit for dMMR/MSI in these tumors and evidence of survival benefit from oxaliplatin-based treatment. Table 2 lists recent studies evaluating predictive utility of dMMR/MSI for adjuvant chemotherapy.

## 5. The Predictive Values of dMMR/MSI for Immunotherapy in Non-Metastatic Colorectal Cancer

MMR deficiency leads to MSI resulting in a high tumor mutational burden (TMB) that is believed to generate highly immunogenic neoantigens that attract cytotoxic T-lymphocyte and Th1 cells to the tumor microenvironment [76]. This may, in part, explain the good prognosis seen in early stage dMMR/MSI CRCs. A recent study of 179 dMMR/MSI CRCs revealed that these tumors had a high rate of mutations in antigen presentation machinery and important immune-modulating pathways [77]. Tumor cells with dMMR/MSI show overexpression of immune checkpoint proteins compared to pMMR/MSS cancers; this may counteract immune surveillance [78]. Together, these features may underlie the dramatic response to immunotherapy that is observed in metastatic dMMR/MSI CRCs.

Le et al. first reported the dramatic and durable response of treatment refractory dMMR/MSI metastatic CRCs to an inhibitor (pembrolizumab) of programmed death protein 1 (PD-1) [79]. The response rate was 50% in the dMMR/MSI population compared to 0% in pMMR/MSS patients. The Checkmate 142 phase 2 study subsequently reported frequent responses to single agent nivolumab, another PD-1 inhibitor [80], or the combination of nivolumab and ipilimumab (a cytotoxic T-lymphocyte-associated protein 4 (CTLA4) inhibitor) in treatment refractory dMMR/MSI metastatic CRC [81]. More recently, single agent pembrolizumab was evaluated in the Keynote 177 study as first-line therapy compared to physician’s choice of chemotherapy. Pembrolizumab treatment demonstrated a dramatic PFS benefit of 16.5 versus 8.2 months (HR 0.60, 95% CI: 0.45–0.80, *p* = 0.0002) in dMMR/MSI metastatic CRCs [82]. Response rate to pembrolizumab monotherapy was 43.8%, and median duration of response was not yet reached at a median follow-up of 28.4 months. Based on these data, pembrolizumab or nivolumab alone or in combination with ipilimumab were FDA-approved for the treatment of dMMR/MSI metastatic CRCs, with pembrolizumab being the only drug approved for first-line treatment. Of note, the FDA approval of pembrolizumab was tumor agnostic and represents the first drug approved irrespective of tumor type if dMMR/MSI is detected.

Since dMMR/MSI has become a verified predictive biomarker for the efficacy of immunotherapy in metastatic CRC, it is reasonable to assume that immunotherapy may also be an effective treatment for earlier stage CRCs as neoadjuvant or adjuvant treatment. The NICHE study enrolled early stage colon cancer patients (21 dMMR/MSI and 20 pMMR/MSS) and treated them with neoadjuvant nivolumab (2 doses on days 1 and 15) plus ipilimumab (1 dose on day 1) followed by surgery within 6 weeks of study enrollment [83]. The primary endpoint of this study was safety and feasibility, and the secondary endpoint was efficacy assessed by histopathological response and changes in T-cell infiltration. Treatment was well tolerated, with only five patients experiencing grade 3–4 treatment-related toxicity. Of 20 dMMR/MSI patients, 19 had a major pathological response (MPR) (defined as ≤10% residual viable tumor in the surgical specimen) of which 12 had pathological complete response (pCR). One patient had a partial pathological response (PPR, defined as ≤50% residual viable tumor in the surgical specimen). Interestingly, four patients with pMMR/MSS disease also had a pathological response (three MPR and one PPR). The impressive pathological responses seen in this study clearly highlight the role of immunotherapy as neoadjuvant treatment for early stage dMMR/MSI CRCs.

Currently, there are two phase III randomized clinical studies that evaluate the role of immunotherapy as adjuvant treatment of dMMR/MSI stage III CRC patients. Studies include the ATOMIC trial that evaluates FOLFOX for 12 cycles alone or combined with the anti-PD-L1 antibody atezolizumab, where the antibody is continued as monotherapy for an additional 6 months. The other trial is known as POLEM and evaluates fluoropyrimidine-based chemotherapy alone or combined with another PD-L1 antibody, avelumab [84,85]. If efficacious, the potential exists for these studies to change standard of care for adjuvant treatment of non-metastatic dMMR/MSI CRCs. Furthermore, these studies may lead to the evaluation of immunotherapy as adjuvant monotherapy or other immunotherapy approaches in this patient population.

## 6. Novel Biomarkers in dMMR/MSI CRCs

Data from immunotherapy trials showed that approximately one-half of patients with dMMR/MSI metastatic CRC respond to immunotherapy and some patients may have disease progression after initial treatment response. Immunotherapy also has a unique side effect profile and is very costly. Therefore, there is an urgent need to find predictive biomarkers to identify the subset of patients with dMMR/MSI who can benefit from immunotherapy.

Tumor mutational burden (TMB) is defined as the number of somatic gene mutations and can be determined by next generation sequencing in tumor tissue [86]. In a study of 22 patients with dMMR/MSI metastatic CRC who were treated with an anti-PD-1 or PD-L1 antibody, TMB had the strongest association with objective tumor response rate [87]. In this study, all 13 dMMR/MSI tumors with a high TMB (cutoff value 37–41 mut/Mb) responded to immunotherapy (median PFS not reached at median follow-up >18 months) while 6/9 patients with low TMB had disease progression on immunotherapy with a median PFS of 2 months for these nine patients. Analysis of the TMB distribution in 18,140 dMMR/MSI metastatic CRCs revealed that a TMB of 37.4 mut/Mb corresponded to 35th percentile in this population. Recent data have shown molecular heterogeneity including TMB within dMMR/MSI CRCs [88]. Analysis of TMB in 1057 dMMR/MSI solid tumors, including CRCs, demonstrated that loss of MLH1/PMS2 was most common (77.2%) as a cause of dMMR and was associated with lower mean TMB than was loss of MSH2/MSH6 (25.0 versus 46.8 mut/Mb). However, further study is needed to confirm these findings and to determine the role of TMB, if any, in responsiveness to immunotherapy.

Binder et al. [89] performed genome-wide DNA and RNA sequencing for HNPCC tumors and classified them into two groups based on the mutational spectrum and microsatellite length. Group 1 is similar to sporadic dMMR/MSI CRC while group 2 is more like MSS CRCs. The clinical implications of this categorization await further study. dMMR/MSI CRCs were also found to be associated with *NTRK* gene rearrangement [90]. In one study, 10 of 13 (76.9%) *NTRK* fusion positive metastatic CRC patients had dMMR/MSI cancer while another study found 2 of 3 (66.7%) NTRK fusion positive colon cancers were dMMR/MSI [90,91]. It is still unknown what the best treatment is for these patients (immunotherapy or NTRK targeted agent).

Studies evaluating the tumor microenvironment and in particular, the density of tumor infiltrating T-lymphocytes (TILs) have shown that TIL densities are prognostic in both dMMR/MSI and pMMR/MSS colon cancers [92]. Furthermore, significantly intertumoral heterogeneity of CD3+ and CD8+ T-cell densities was observed in dMMR/MSI CRCs and these data suggest that tumors with lower T-cell densities may contribute to immunotherapy resistance [93].

β2-microglobulin (B2M) is part of the major histocompatibility complex I (MHC-I)/human leukocyte antigen class 1 (HLA-1) and is critical for antigen presentation [94]. Studies have shown an association of *β2-microglobulin* (*B2M*) mutation with tumor metastases in dMMR/MSI CRCs. In this regard, Kloor et al. evaluated B2M mutation status in 104 dMMR/MSI CRC patients and found *B2M* mutation was only detected in localized disease [95]. Barrow et al. investigated B2M mutation and protein expression in 229 stage II colon cancer patients (121 dMMR/MSI cases) in the QUASAR study [96]. The investigators detected *B2M* mutations in 32% (39/121 cases) of dMMR/MSI tumors and none of these patients had tumor recurrence while 18% of *B2M* wild-type tumors developed disease recurrence. Other studies also reported similar results [97,98] and together, indicate that mutation in *B2M* is associated with suppression of tumor metastasis.

Fragments of DNA are shed into the bloodstream from dividing cells during cell proliferation or cell death and are referred to as cell free DNA (cfDNA). In patients with cancer, a fraction of the cfDNA is tumor-derived and is known as circulating tumor DNA (ctDNA) [99,100]. Recent studies have demonstrated the promise of detectable ctDNA to indicate minimal residual disease with increased risk of recurrence following curative-intent surgery in patients with non-metastatic CRC [101,102,103]. However, data are lacking as to how to optimally manage patients with positive ctDNA in the adjuvant setting, although it can be agreed that such patients need adjuvant treatment given their high risk of recurrence. Currently, a phase 2 study is investigating the role of adjuvant pembrolizumab in patients with positive ctDNA after resection of their dMMR/MSI solid tumors (NCT03832569).

## 7. Conclusions

Approximately two-thirds of dMMR/MSI CRCs are sporadics that generally result from epigenetic inactivation of the *MLH1* gene promoter. The other one-third of tumors are found in association with Lynch Syndrome that arises from a heritable germline mutation in an MMR gene. dMMR/MSI is associated with favorable prognosis in early stage CRCs, and evidence indicates that adjuvant chemotherapy is not beneficial in stage II dMMR/MSI colon cancer patients. The prognostic value of dMMR/MSI in stage III disease is less robust compared to stage II patients, and these patients should receive standard adjuvant chemotherapy with a combination of fluoropyrimidine and oxaliplatin.

dMMR/MSI is a predictive biomarker for favorable response to immune checkpoint inhibitors in patients with metastatic disease, although the role of immunotherapy in earlier stage CRCs is under active investigation in large randomized studies. Studies examining molecular heterogeneity as well as intertumoral heterogeneity of the tumor microenvironment within the dMMR/MSI tumors provide an increased understanding of de novo or acquired resistance to immunotherapy in these tumors and may lead to the identification of new predictive biomarkers.

Major implications for clinicians:

- Stage II dMMR/MSI colon cancers have a favorable prognosis and no adjuvant treatment is recommended.
- The prognostic advantage of dMMR/MSI is attenuated in stage III vs. stage II colon cancers, and oxaliplatin-based adjuvant treatment is recommended.

Major open questions:

- The role of adjuvant immunotherapy for stage III colorectal cancer (alone or combined with chemotherapy).
- The potential for molecular biomarkers to guide the use of adjuvant treatment for dMMR/MSI stage II/III colorectal cancer patients.

## Figures and Tables

**Table 1:** Recent studies evaluating the prognostic value of dMMR/MSI in colorectal cancer.

Study Name	Year of Publication	Disease Stage	Patient Number	dMMR/MSI Patients	Endpoint	HR (95% CI)	*p* Value
QUASAR study [50] *	2011	II	636	167	RR	0.44 (0.29–0.67)	<0.001
Pooled analysis [51]	2010	II, III	1027	165	DFS	0.51 (0.29–0.89	0.009
					OS	0.47 (0.26–0.83)	0.004
ACCENT analysis [52]	2014	II	2270	524	OS	0.27 (0.10–0.74)	0.01
					TTR	0.27 (0.10–0.75)	0.01
Pooled analysis [53]	2010	II, III	2141	344	DFS	0.80 (0.64–0.99)	0.035
					OS	0.79 (0.64–0.99)	0.031
					TTR	0.72 (0.56–0.91)	0.005
PETACC3 [54]	2012	II, III	1404	210	RFS	0.54 (0.37–0.81)	0.003
					OS	0.43 (0.27–0.70)	0.001
NSABP C-07/08 [55]	2012	II, III	2299	207	TTR	0.48 (0.33–0.70)	0.0001
					OS	0.64 (0.46–0.89)	<0.01
					SAR	1.60 (1.07–2.41)	0.02
ACCENT analysis [56]	2019	III	2630	271	SAR	0.82 (0.69–0.98)	0.029
NCCTG N0147 [57]	2013	III	2580	314	DFS	0.82 (0.64–1.07)	0.14
					DFS Proximal	0.71 (0.53–0.94)	0.053
					DFS Distal	1.71 (0.99–2.95)	0.056
PETACC3 [58]	2015	II	309	86	RFS	0.26 (0.10–0.65)	NR
					OS	0.16 (0.04–0.64)	NR
		III	755	104	RFS	0.67 (0.46–0.99)	NR
					OS	0.70 (0.44–1.09)	NR
PETACC-8 and NCCTG N0147 [59] **	2017	III	4411	477	TTR *KRAS* mutant	1.04 (0.57–1.90)	0.91
					OS *KRAS* mutant	1.07 0.57–2.02)	0.84
					TTR *BRAF*^V600E^	0.94 (0.58–1.51)	0.80
					OS *BRAF*^V600E^	1.26 (0.78–2.04)	0.35
Systemic review and meta-analysis [60]	2019	II, III	17065	2337	OS	0.74 (0.68–0.82)	<0.001
			23195	3264	DFS	0.67 (0.59–0.75)	<0.001
		III	7481	907	OS	0.71 (0.63–0.81)	<0.001
			10714	1198	DFS	0.69 (0.60–0.80)	<0.001
Meta-analysis [61]	2019	III RCT	NR	NR	OS	0.96 (0.75–1.23)	NR
					DFS	0.83 (0.65–1.07)	NR
		III non-RCT	NR	NR	OS	0.89 (0.62–1.28)	NR
					DFS	0.83 (0.65–1.07)	NR
					DSS	1.07 (0.68–1.69)	NR

Abbreviations: HR: hazard ratio; CI: confidence interval; QUASAR: Quick and Simple and Reliable; RR: recurrence rate; ACCENT: Adjuvant Colon Cancer Endpoint database; OS: overall survival; TTR: time to recurrence; DFS: disease free survival; PETACC: Pan-European Trial in Alimentary Tract Cancer; RFS: recurrence free survival; NSABP: National Surgical Adjuvant Breast and Bowel Project; SAR: survival after recurrence; NCCTG: North Central Cancer Treatment Group; RCT: randomized controlled trials; DSS: disease specific survival. * Only reported the right-sided colon cancer. ** Only listed analysis for dMMR/MSI population

**Table 2:** Recent studies evaluating the predictive value of dMMR/MSI in colorectal cancer.

Study Details	Year Publication	Disease Stage	Patient Number	dMMR/MSI pts	Adjuvant Chemo	Endpoint	HR (95% CI)	*p* Value
Pooled analysis [68]	2003	II, III	570	95	5-FU	OS (no adjuvant)	0.31 (0.14–0.72)	0.004
						OS (with adjuvant)	1.07 (0.62–1.86)	0.80
		II	312	58	5-FU	OS (with adjuvant)	3.28 (0.86–12.48)	NR
		III	258	37		OS (with adjuvant)	1.42 (0.36–5.56)	NR
Pooled analysis [51]	2010	II and III	457	70	5-FU	DFS (with adjuvant)	1.10 (0.42–2.91)	0.85
			1027	165		DFS, stage II (with adjuvant)	2.30 (0.85–6.24)	0.09
						DFS stage III (with adjuvant)	1.01 (0.41–2.51)	0.98
						OS stage II (with adjuvant)	2.95 (1.02–8.94)	0.04
Korean study [69]	2015	II	860	126	5-FU	DFS (with adjuvant)	0.557	0.254
						OS (with adjuvant)	0.288	0.033
QUASAR [50]	2011	II	1913	218	5-FU	OR (for recurrence)	0.81 (0.29–2.22)	NR
Pooled analysis [53]	2011	II and III	2141	344	5-FU	5-year recurrence rate *	NR	0.044
						Distant recurrence *	NR	0.011
						Liver recurrence *	NR	0.005
Retrospective study [72]	2010	III	233	32	FOLFOX vs. 5-FU/LV	DFS	0.17 (0.04–0.68)	0.01
MOSAIC subgroup study [73]	2015	II and III	1008	95	FOLFOX vs. 5-FU/LV	DFS	0.48 (0.21–1.12)	0.088
						OS	0.41 (0.16–1.07)	0.069
AGEO study [74]	2016	II and III	433	433	FOLFOX vs. 5-FU/LV vs. surgery alone	DFS **	0.41 (0.19–0.87)	0.02

Abbreviations: HR: hazard ratio; CI: confidence interval; 5-FU: Fluorouracil; OS: overall survival; NR: not reported; DFS: disease free survival; QUASAR: Quick and Simple and Reliable; OR: odds ratio; FOLFOX: Fluorouracil, Leucovorin, and oxaliplatin. * Only listed stage III cancers (1183 patients with 180 dMMR/MSI cases). ** Only for stage III disease

## References

[1] GBD 2017 Colorectal Cancer Collaborators (2019). The global, regional, and national burden of colorectal cancer and its attributable risk factors in 195 countries and territories, 1990–2017: A systematic analysis for the Global Burden of Disease Study 2017. Lancet Gastroenterol. Hepatol..

[2] Siegel R.L., Miller K.D., Goding Sauer A., Fedewa S.A., Butterly L.F., Anderson J.C., Cercek A., Smith R.A., Jemal A. (2020). Colorectal cancer statistics, 2020. CA Cancer J. Clin..

[3] Jenkins M.A., Hayashi S., O’Shea A.M., Burgart L.J., Smyrk T.C., Shimizu D., Waring P.M., Ruszkiewicz A.R., Pollett A.F., Redston M. (2007). Pathology features in Bethesda guidelines predict colorectal cancer microsatellite instability: A population-based study. Gastroenterology.

[4] Bessa X., Alenda C., Paya A., Alvarez C., Iglesias M., Seoane A., Dedeu J.M., Abuli A., Ilzarbe L., Navarro G. (2011). Validation microsatellite path score in a population-based cohort of patients with colorectal cancer. J. Clin. Oncol..

[5] Cancer Genome Atlas N. (2012). Comprehensive molecular characterization of human colon and rectal cancer. Nature.

[6] Schwitalle Y., Kloor M., Eiermann S., Linnebacher M., Kienle P., Knaebel H.P., Tariverdian M., Benner A., von Knebel Doeberitz M. (2008). Immune response against frameshift-induced neopeptides in HNPCC patients and healthy HNPCC mutation carriers. Gastroenterology.

[7] Popat S., Hubner R., Houlston R.S. (2005). Systematic review of microsatellite instability and colorectal cancer prognosis. J. Clin. Oncol..

[8] Zaanan A., Meunier K., Sangar F., Flejou J.F., Praz F. (2011). Microsatellite instability in colorectal cancer: From molecular oncogenic mechanisms to clinical implications. Cell. Oncol..

[9] Gemayel R., Cho J., Boeynaems S., Verstrepen K.J. (2012). Beyond junk-variable tandem repeats as facilitators of rapid evolution of regulatory and coding sequences. Genes.

[10] Lawson M.J., Zhang L. (2006). Distinct patterns of SSR distribution in the Arabidopsis thaliana and rice genomes. Genome Biol..

[11] Vieira M.L., Santini L., Diniz A.L., Cde M.F. (2016). Microsatellite markers: What they mean and why they are so useful. Genet. Mol. Biol..

[12] Ionov Y., Peinado M.A., Malkhosyan S., Shibata D., Perucho M. (1993). Ubiquitous somatic mutations in simple repeated sequences reveal a new mechanism for colonic carcinogenesis. Nature.

[13] Thibodeau S.N., Bren G., Schaid D. (1993). Microsatellite instability in cancer of the proximal colon. Science.

[14] Peltomaki P., Lothe R.A., Aaltonen L.A., Pylkkanen L., Nystrom-Lahti M., Seruca R., David L., Holm R., Ryberg D., Haugen A. (1993). Microsatellite instability is associated with tumors that characterize the hereditary non-polyposis colorectal carcinoma syndrome. Cancer Res..

[15] Aaltonen L.A., Peltomaki P., Leach F.S., Sistonen P., Pylkkanen L., Mecklin J.P., Jarvinen H., Powell S.M., Jen J., Hamilton S.R. (1993). Clues to the pathogenesis of familial colorectal cancer. Science.

[16] Lothe R.A., Peltomaki P., Meling G.I., Aaltonen L.A., Nystrom-Lahti M., Pylkkanen L., Heimdal K., Andersen T.I., Moller P., Rognum T.O. (1993). Genomic instability in colorectal cancer: Relationship to clinicopathological variables and family history. Cancer Res..

[17] Bronner C.E., Baker S.M., Morrison P.T., Warren G., Smith L.G., Lescoe M.K., Kane M., Earabino C., Lipford J., Lindblom A. (1994). Mutation in the DNA mismatch repair gene homologue hMLH1 is associated with hereditary non-polyposis colon cancer. Nature.

[18] Papadopoulos N., Nicolaides N.C., Wei Y.F., Ruben S.M., Carter K.C., Rosen C.A., Haseltine W.A., Fleischmann R.D., Fraser C.M., Adams M.D. (1994). Mutation of a mutL homolog in hereditary colon cancer. Science.

[19] Nicolaides N.C., Papadopoulos N., Liu B., Wei Y.F., Carter K.C., Ruben S.M., Rosen C.A., Haseltine W.A., Fleischmann R.D., Fraser C.M. (1994). Mutations of two PMS homologues in hereditary nonpolyposis colon cancer. Nature.

[20] Miyaki M., Konishi M., Tanaka K., Kikuchi-Yanoshita R., Muraoka M., Yasuno M., Igari T., Koike M., Chiba M., Mori T. (1997). Germline mutation of MSH6 as the cause of hereditary nonpolyposis colorectal cancer. Nat. Genet..

[21] Ligtenberg M.J., Kuiper R.P., Geurts van Kessel A., Hoogerbrugge N. (2013). EPCAM deletion carriers constitute a unique subgroup of Lynch syndrome patients. Fam. Cancer.

[22] Hampel H., Frankel W.L., Martin E., Arnold M., Khanduja K., Kuebler P., Nakagawa H., Sotamaa K., Prior T.W., Westman J. (2005). Screening for the Lynch syndrome (hereditary nonpolyposis colorectal cancer). N. Engl. J. Med..

[23] Ward R., Meagher A., Tomlinson I., O’Connor T., Norrie M., Wu R., Hawkins N. (2001). Microsatellite instability and the clinicopathological features of sporadic colorectal cancer. Gut.

[24] Wang L., Cunningham J.M., Winters J.L., Guenther J.C., French A.J., Boardman L.A., Burgart L.J., McDonnell S.K., Schaid D.J., Thibodeau S.N. (2003). BRAF mutations in colon cancer are not likely attributable to defective DNA mismatch repair. Cancer Res..

[25] Sinicrope F.A., Rego R.L., Halling K.C., Foster N., Sargent D.J., La Plant B., French A.J., Laurie J.A., Goldberg R.M., Thibodeau S.N. (2006). Prognostic impact of microsatellite instability and DNA ploidy in human colon carcinoma patients. Gastroenterology.

[26] Toyota M., Ahuja N., Ohe-Toyota M., Herman J.G., Baylin S.B., Issa J.P. (1999). CpG island methylator phenotype in colorectal cancer. Proc. Natl. Acad. Sci. USA.

[27] Issa J.P. (2004). CpG island methylator phenotype in cancer. Nat. Rev. Cancer.

[28] Weisenberger D.J., Siegmund K.D., Campan M., Young J., Long T.I., Faasse M.A., Kang G.H., Widschwendter M., Weener D., Buchanan D. (2006). CpG island methylator phenotype underlies sporadic microsatellite instability and is tightly associated with BRAF mutation in colorectal cancer. Nat. Genet..

[29] Hinoue T., Weisenberger D.J., Lange C.P., Shen H., Byun H.M., Van Den Berg D., Malik S., Pan F., Noushmehr H., van Dijk C.M. (2012). Genome-scale analysis of aberrant DNA methylation in colorectal cancer. Genome Res..

[30] Rodriguez-Soler M., Perez-Carbonell L., Guarinos C., Zapater P., Castillejo A., Barbera V.M., Juarez M., Bessa X., Xicola R.M., Clofent J. (2013). Risk of cancer in cases of suspected lynch syndrome without germline mutation. Gastroenterology.

[31] Mensenkamp A.R., Vogelaar I.P., van Zelst-Stams W.A., Goossens M., Ouchene H., Hendriks-Cornelissen S.J., Kwint M.P., Hoogerbrugge N., Nagtegaal I.D., Ligtenberg M.J. (2014). Somatic mutations in MLH1 and MSH2 are a frequent cause of mismatch-repair deficiency in Lynch syndrome-like tumors. Gastroenterology.

[32] Haraldsdottir S., Hampel H., Tomsic J., Frankel W.L., Pearlman R., de la Chapelle A., Pritchard C.C. (2014). Colon and endometrial cancers with mismatch repair deficiency can arise from somatic, rather than germline, mutations. Gastroenterology.

[33] Cohen S.A., Turner E.H., Beightol M.B., Jacobson A., Gooley T.A., Salipante S.J., Haraldsdottir S., Smith C., Scroggins S., Tait J.F. (2016). Frequent PIK3CA mutations in colorectal and endometrial tumors with 2 or more somatic mutations in mismatch repair genes. Gastroenterology.

[34] Duval A., Hamelin R. (2002). Mutations at coding repeat sequences in mismatch repair-deficient human cancers: Toward a new concept of target genes for instability. Cancer Res..

[35] Lynch H.T., Lynch J.F., Attard T.A. (2009). Diagnosis and management of hereditary colorectal cancer syndromes: Lynch syndrome as a model. CMAJ.

[36] Lynch H.T., Boland C.R., Gong G., Shaw T.G., Lynch P.M., Fodde R., Lynch J.F., de la Chapelle A. (2006). Phenotypic and genotypic heterogeneity in the Lynch syndrome: Diagnostic, surveillance and management implications. Eur. J. Hum. Genet..

[37] Bui Q.M., Lin D., Ho W. (2017). Approach to lynch syndrome for the gastroenterologist. Dig. Dis. Sci..

[38] Malkhosyan S.R., Yamamoto H., Piao Z., Perucho M. (2000). Late onset and high incidence of colon cancer of the mutator phenotype with hypermethylated hMLH1 gene in women. Gastroenterology.

[39] Win A.K., Buchanan D.D., Rosty C., MacInnis R.J., Dowty J.G., Dite G.S., Giles G.G., Southey M.C., Young J.P., Clendenning M. (2015). Role of tumour molecular and pathology features to estimate colorectal cancer risk for first-degree relatives. Gut.

[40] Parry S., Win A.K., Parry B., Macrae F.A., Gurrin L.C., Church J.M., Baron J.A., Giles G.G., Leggett B.A., Winship I. (2011). Metachronous colorectal cancer risk for mismatch repair gene mutation carriers: The advantage of more extensive colon surgery. Gut.

[41] Sepulveda A.R., Hamilton S.R., Allegra C.J., Grody W., Cushman-Vokoun A.M., Funkhouser W.K., Kopetz S.E., Lieu C., Lindor N.M., Minsky B.D. (2017). Molecular biomarkers for the evaluation of colorectal cancer: Guideline from the American Society for Clinical Pathology, College of American Pathologists, Association for Molecular Pathology, and the American Society of Clinical Oncology. J. Clin. Oncol..

[42] Loughrey M.B., McGrath J., Coleman H.G., Bankhead P., Maxwell P., McGready C., Bingham V., Humphries M.P., Craig S.G., McQuaid S. (2020). Identifying mismatch repair-deficient colon cancer: Near-perfect concordance between immunohistochemistry and microsatellite instability testing in a large, population-based series. Histopathology.

[43] Cicek M.S., Lindor N.M., Gallinger S., Bapat B., Hopper J.L., Jenkins M.A., Young J., Buchanan D., Walsh M.D., Le Marchand L. (2011). Quality assessment and correlation of microsatellite instability and immunohistochemical markers among population- and clinic-based colorectal tumors results from the Colon Cancer Family Registry. J. Mol. Diagn..

[44] Evrard C., Tachon G., Randrian V., Karayan-Tapon L., Tougeron D. (2019). Microsatellite instability: Diagnosis, heterogeneity, discordance, and clinical impact in colorectal cancer. Cancers.

[45] Hall M.J., Gowen K., Sanford E.M., Elvin J.A., Ali S.M., Kaczmar J., White E., Malboeuf C., Ross J.S., Miller V.A. (2016). Evaluation of microsatellite instability (MSI) status in 11,573 diverse solid tumors using comprehensive genomic profiling (CGP). J. Clin. Oncol..

[46] Middha S., Zhang L., Nafa K., Jayakumaran G., Wong D., Kim H.R., Sadowska J., Berger M.F., Delair D.F., Shia J. (2017). Reliable pan-cancer microsatellite instability assessment by using targeted next-generation sequencing data. JCO Precis. Oncol..

[47] Pabla S., Andreas J., Lenzo F.L., Burgher B., Hagen J., Giamo V., Nesline M.K., Wang Y., Gardner M., Conroy J.M. (2019). Development and analytical validation of a next-generation sequencing based microsatellite instability (MSI) assay. Oncotarget.

[48] Willis J., Lefterova M.I., Artyomenko A., Kasi P.M., Nakamura Y., Mody K., Catenacci D.V.T., Fakih M., Barbacioru C., Zhao J. (2019). Validation of microsatellite instability detection using a comprehensive plasma-based genotyping panel. Clin. Cancer Res..

[49] Koopman M., Kortman G.A., Mekenkamp L., Ligtenberg M.J., Hoogerbrugge N., Antonini N.F., Punt C.J., van Krieken J.H. (2009). Deficient mismatch repair system in patients with sporadic advanced colorectal cancer. Br. J. Cancer.

[50] Hutchins G., Southward K., Handley K., Magill L., Beaumont C., Stahlschmidt J., Richman S., Chambers P., Seymour M., Kerr D. (2011). Value of mismatch repair, KRAS, and BRAF mutations in predicting recurrence and benefits from chemotherapy in colorectal cancer. J. Clin. Oncol..

[51] Sargent D.J., Marsoni S., Monges G., Thibodeau S.N., Labianca R., Hamilton S.R., French A.J., Kabat B., Foster N.R., Torri V. (2010). Defective mismatch repair as a predictive marker for lack of efficacy of fluorouracil-based adjuvant therapy in colon cancer. J. Clin. Oncol..

[52] Sargent D.J., Shi Q., Yothers G., Tejpar S., Bertagnolli M.M., Thibodeau S.N., Andre T., Labianca R., Gallinger S., Hamilton S.R. (2014). Prognostic impact of deficient mismatch repair (dMMR) in 7,803 stage II/III colon cancer (CC) patients (pts): A pooled individual pt data analysis of 17 adjuvant trials in the ACCENT database. J. Clin. Oncol..

[53] Sinicrope F.A., Foster N.R., Thibodeau S.N., Marsoni S., Monges G., Labianca R., Yothers G., Allegra C., Moore M.J., Gallinger S. (2011). DNA mismatch repair status and colon cancer recurrence and survival in clinical trials of 5-fluorouracil-based adjuvant therapy. J. Natl. Cancer Inst..

[54] Roth A.D., Delorenzi M., Tejpar S., Yan P., Klingbiel D., Fiocca R., d’Ario G., Cisar L., Labianca R., Cunningham D. (2012). Integrated analysis of molecular and clinical prognostic factors in stage II/III colon cancer. J. Natl. Cancer Inst..

[55] Gavin P.G., Colangelo L.H., Fumagalli D., Tanaka N., Remillard M.Y., Yothers G., Kim C., Taniyama Y., Kim S.I., Choi H.J. (2012). Mutation profiling and microsatellite instability in stage II and III colon cancer: An assessment of their prognostic and oxaliplatin predictive value. Clin. Cancer Res..

[56] Taieb J., Shi Q., Pederson L., Alberts S., Wolmark N., Van Cutsem E., de Gramont A., Kerr R., Grothey A., Lonardi S. (2019). Prognosis of microsatellite instability and/or mismatch repair deficiency stage III colon cancer patients after disease recurrence following adjuvant treatment: Results of an ACCENT pooled analysis of seven studies. Ann. Oncol..

[57] Sinicrope F.A., Mahoney M.R., Smyrk T.C., Thibodeau S.N., Warren R.S., Bertagnolli M.M., Nelson G.D., Goldberg R.M., Sargent D.J., Alberts S.R. (2013). Prognostic impact of deficient dna mismatch repair in patients with stage III colon cancer from a randomized trial of FOLFOX-based adjuvant chemotherapy. J. Clin. Oncol..

[58] Klingbiel D., Saridaki Z., Roth A.D., Bosman F.T., Delorenzi M., Tejpar S. (2015). Prognosis of stage II and III colon cancer treated with adjuvant 5-fluorouracil or FOLFIRI in relation to microsatellite status: Results of the PETACC-3 trial. Ann. Oncol..

[59] Taieb J., Le Malicot K., Shi Q., Penault-Llorca F., Bouche O., Tabernero J., Mini E., Goldberg R.M., Folprecht G., Luc Van Laethem J. (2017). Prognostic value of BRAF and KRAS mutations in MSI and MSS stage III colon cancer. J. Natl. Cancer Inst..

[60] Deng Z., Qin Y., Wang J., Wang G., Lang X., Jiang J., Xie K., Zhang W., Xu H., Shu Y. (2020). Prognostic and predictive role of DNA mismatch repair status in stage II-III colorectal cancer: A systematic review and meta-analysis. Clin. Genet..

[61] Wang B., Li F., Zhou X., Ma Y., Fu W. (2019). Is microsatellite instability-high really a favorable prognostic factor for advanced colorectal cancer? A meta-analysis. World J. Surg. Oncol..

[62] Buckowitz A., Knaebel H.P., Benner A., Blaker H., Gebert J., Kienle P., von Doeberitz K.M., Kloor M. (2005). Microsatellite instability in colorectal cancer is associated with local lymphocyte infiltration and low frequency of distant metastases. Br. J. Cancer.

[63] Venderbosch S., Nagtegaal I.D., Maughan T.S., Smith C.G., Cheadle J.P., Fisher D., Kaplan R., Quirke P., Seymour M.T., Richman S.D. (2014). Mismatch repair status and BRAF mutation status in metastatic colorectal cancer patients: A pooled analysis of the CAIRO, CAIRO2, COIN, and FOCUS studies. Clin. Cancer Res..

[64] Hemminki A., Mecklin J.P., Jarvinen H., Aaltonen L.A., Joensuu H. (2000). Microsatellite instability is a favorable prognostic indicator in patients with colorectal cancer receiving chemotherapy. Gastroenterology.

[65] Elsaleh H., Powell B., Soontrapornchai P., Joseph D., Goria F., Spry N., Iacopetta B. (2000). p53 gene mutation, microsatellite instability and adjuvant chemotherapy: Impact on survival of 388 patients with Dukes’ C colon carcinoma. Oncology.

[66] Elsaleh H., Joseph D., Grieu F., Zeps N., Spry N., Iacopetta B. (2000). Association of tumour site and sex with survival benefit from adjuvant chemotherapy in colorectal cancer. Lancet.

[67] Elsaleh H., Powell B., McCaul K., Grieu F., Grant R., Joseph D., Iacopetta B. (2001). p53 alteration and microsatellite instability have predictive value for survival benefit from chemotherapy in stage III colorectal carcinoma. Clin. Cancer Res..

[68] Ribic C.M., Sargent D.J., Moore M.J., Thibodeau S.N., French A.J., Goldberg R.M., Hamilton S.R., Laurent-Puig P., Gryfe R., Shepherd L.E. (2003). Tumor microsatellite-instability status as a predictor of benefit from fluorouracil-based adjuvant chemotherapy for colon cancer. N. Engl. J. Med..

[69] Kim J.E., Hong Y.S., Kim H.J., Kim K.P., Lee J.L., Park S.J., Lim S.B., Park I.J., Kim C.W., Yoon Y.S. (2015). Defective mismatch repair status was not associated with DFS and OS in stage II colon cancer treated with adjuvant chemotherapy. Ann. Surg. Oncol..

[70] Bertagnolli M.M., Redston M., Compton C.C., Niedzwiecki D., Mayer R.J., Goldberg R.M., Colacchio T.A., Saltz L.B., Warren R.S. (2011). Microsatellite instability and loss of heterozygosity at chromosomal location 18q: Prospective evaluation of biomarkers for stages II and III colon cancer—A study of CALGB 9581 and 89803. J. Clin. Oncol..

[71] Andre T., Boni C., Mounedji-Boudiaf L., Navarro M., Tabernero J., Hickish T., Topham C., Zaninelli M., Clingan P., Bridgewater J. (2004). Oxaliplatin, fluorouracil, and leucovorin as adjuvant treatment for colon cancer. N. Engl. J. Med..

[72] Zaanan A., Cuilliere-Dartigues P., Guilloux A., Parc Y., Louvet C., de Gramont A., Tiret E., Dumont S., Gayet B., Validire P. (2010). Impact of p53 expression and microsatellite instability on stage III colon cancer disease-free survival in patients treated by 5-fluorouracil and leucovorin with or without oxaliplatin. Ann. Oncol..

[73] Andre T., de Gramont A., Vernerey D., Chibaudel B., Bonnetain F., Tijeras-Raballand A., Scriva A., Hickish T., Tabernero J., Van Laethem J.L. (2015). Adjuvant fluorouracil, leucovorin, and oxaliplatin in stage II to III colon cancer: Updated 10-year survival and outcomes according to BRAF mutation and mismatch repair status of the MOSAIC study. J. Clin. Oncol..

[74] Tougeron D., Mouillet G., Trouilloud I., Lecomte T., Coriat R., Aparicio T., Des Guetz G., Lecaille C., Artru P., Sickersen G. (2016). Efficacy of adjuvant chemotherapy in colon cancer with microsatellite instability: A large multicenter AGEO study. J. Natl. Cancer Inst..

[75] Cohen R., Taieb J., Fiskum J., Yothers G., Goldberg R., Yoshino T., Alberts S., Allegra C., de Gramont A., Seitz J.F. (2020). Microsatellite Instability in Patients With Stage III Colon Cancer Receiving Fluoropyrimidine With or Without Oxaliplatin: An ACCENT Pooled Analysis of 12 Adjuvant Trials. J. Clin. Oncol..

[76] Rizvi N.A., Hellmann M.D., Snyder A., Kvistborg P., Makarov V., Havel J.J., Lee W., Yuan J., Wong P., Ho T.S. (2015). Cancer immunology. Mutational landscape determines sensitivity to PD-1 blockade in non-small cell lung cancer. Science.

[77] Grasso C.S., Giannakis M., Wells D.K., Hamada T., Mu X.J., Quist M., Nowak J.A., Nishihara R., Qian Z.R., Inamura K. (2018). Genetic mechanisms of immune evasion in colorectal cancer. Cancer Discov..

[78] Llosa N.J., Cruise M., Tam A., Wicks E.C., Hechenbleikner E.M., Taube J.M., Blosser R.L., Fan H., Wang H., Luber B.S. (2015). The vigorous immune microenvironment of microsatellite instable colon cancer is balanced by multiple counter-inhibitory checkpoints. Cancer Discov..

[79] Le D.T., Uram J.N., Wang H., Bartlett B.R., Kemberling H., Eyring A.D., Skora A.D., Luber B.S., Azad N.S., Laheru D. (2015). PD-1 blockade in tumors with mismatch-repair deficiency. N. Engl. J. Med..

[80] Overman M.J., McDermott R., Leach J.L., Lonardi S., Lenz H.J., Morse M.A., Desai J., Hill A., Axelson M., Moss R.A. (2017). Nivolumab in patients with metastatic DNA mismatch repair-deficient or microsatellite instability-high colorectal cancer (CheckMate 142): An open-label, multicentre, phase 2 study. Lancet Oncol..

[81] Overman M.J., Lonardi S., Wong K.Y.M., Lenz H.J., Gelsomino F., Aglietta M., Morse M.A., Van Cutsem E., McDermott R., Hill A. (2018). Durable clinical benefit with nivolumab plus ipilimumab in DNA mismatch repair-deficient/microsatellite instability-high metastatic colorectal cancer. J. Clin. Oncol..

[82] Andre T., Shiu K.K., Kim T.W., Jensen B.V., Jensen L.H., Punt C., Smith D., Garcia-Carbonero R., Benavides M., Gibbs P. (2020). Pembrolizumab in microsatellite-instability-high advanced colorectal cancer. N. Engl. J. Med..

[83] Chalabi M., Fanchi L.F., Dijkstra K.K., Van den Berg J.G., Aalbers A.G., Sikorska K., Lopez-Yurda M., Grootscholten C., Beets G.L., Snaebjornsson P. (2020). Neoadjuvant immunotherapy leads to pathological responses in MMR-proficient and MMR-deficient early-stage colon cancers. Nat. Med..

[84] Sinicrope F.A., Ou F.-S., Zemla T., Nixon A.B., Mody K., Levasseur A., Dueck A.C., Dhanarajan A.R., Lieu C.H., Cohen D.J. (2019). Randomized trial of standard chemotherapy alone or combined with atezolizumab as adjuvant therapy for patients with stage III colon cancer and deficient mismatch repair (ATOMIC, Alliance A021502). J. Clin. Oncol..

[85] Lau D., Cunningham D., Gillbanks A., Crux R., Powell R., Kalaitzaki E., Annels N.E., Sclafani F., Gerlinger M., Chau I. (2019). POLEM: Avelumab plus fluoropyrimidine-based chemotherapy as adjuvant treatment for stage III dMMR or POLE exonuclease domain mutant colon cancer—A phase III randomized study. J. Clin. Oncol..

[86] Sha D., Jin Z., Budczies J., Kluck K., Stenzinger A., Sinicrope F.A. (2020). Tumor mutational burden as a predictive biomarker in solid tumors. Cancer Discov..

[87] Schrock A.B., Ouyang C., Sandhu J., Sokol E., Jin D., Ross J.S., Miller V.A., Lim D., Amanam I., Chao J. (2019). Tumor mutational burden is predictive of response to immune checkpoint inhibitors in MSI-high metastatic colorectal cancer. Ann. Oncol..

[88] Salem M.E., Bodor J.N., Puccini A., Xiu J., Goldberg R.M., Grothey A., Korn W.M., Shields A.F., Worrilow W.M., Kim E.S. (2020). Relationship between MLH1, PMS2, MSH2 and MSH6 gene-specific alterations and tumor mutational burden in 1057 microsatellite instability-high solid tumors. Int. J. Cancer.

[89] Binder H., Hopp L., Schweiger M.R., Hoffmann S., Juhling F., Kerick M., Timmermann B., Siebert S., Grimm C., Nersisyan L. (2017). Genomic and transcriptomic heterogeneity of colorectal tumours arising in Lynch syndrome. J. Pathol..

[90] Pietrantonio F., Di Nicolantonio F., Schrock A.B., Lee J., Tejpar S., Sartore-Bianchi A., Hechtman J.F., Christiansen J., Novara L., Tebbutt N. (2017). ALK, ROS1, and NTRK rearrangements in metastatic colorectal cancer. J. Natl. Cancer Inst..

[91] Okamura R., Boichard A., Kato S., Sicklick J.K., Bazhenova L., Kurzrock R. (2018). Analysis of NTRK alterations in pan-cancer adult and pediatric malignancies: Implications for NTRK-targeted therapeutics. JCO Precis. Oncol..

[92] Idos G.E., Kwok J., Bonthala N., Kysh L., Gruber S.B., Qu C.X. (2020). The prognostic implications of tumor infiltrating lymphocytes in colorectal cancer: A systematic review and Meta-analysis. Sci. Rep..

[93] Yoon H.H., Shi Q., Heying E.N., Muranyi A., Bredno J., Ough F., Djalilvand A., Clements J., Bowermaster R., Liu W.W. (2019). Intertumoral heterogeneity of CD3(+) and CD8(+) T-cell densities in the microenvironment of DNA mismatch-repair-deficient colon cancers: Implications for prognosis. Clin. Cancer Res..

[94] Argyropoulos C.P., Chen S.S., Ng Y.H., Roumelioti M.E., Shaffi K., Singh P.P., Tzamaloukas A.H. (2017). Rediscovering Beta-2 microglobulin as a biomarker across the spectrum of kidney diseases. Front. Med..

[95] Kloor M., Michel S., Buckowitz B., Ruschoff J., Buttner R., Holinski-Feder E., Dippold W., Wagner R., Tariverdian M., Benner A. (2007). Beta2-microglobulin mutations in microsatellite unstable colorectal tumors. Int. J. Cancer.

[96] Barrow P., Richman S.D., Wallace A.J., Handley K., Hutchins G.G.A., Kerr D., Magill L., Evans D.G., Gray R., Quirke P. (2019). Confirmation that somatic mutations of beta-2 microglobulin correlate with a lack of recurrence in a subset of stage II mismatch repair deficient colorectal cancers from the QUASAR trial. Histopathology.

[97] Koelzer V.H., Baker K., Kassahn D., Baumhoer D., Zlobec I. (2012). Prognostic impact of beta-2-microglobulin expression in colorectal cancers stratified by mismatch repair status. J. Clin. Pathol..

[98] Tikidzhieva A., Benner A., Michel S., Formentini A., Link K.H., Dippold W., Doeberitz M.V., Kornmann M., Kloor M. (2012). Microsatellite instability and Beta2-Microglobulin mutations as prognostic markers in colon cancer: Results of the FOGT-4 trial. Br. J. Cancer.

[99] Stroun M., Lyautey J., Lederrey C., Olson-Sand A., Anker P. (2001). About the possible origin and mechanism of circulating DNA apoptosis and active DNA release. Clin. Chim. Acta.

[100] Bettegowda C., Sausen M., Leary R.J., Kinde I., Wang Y., Agrawal N., Bartlett B.R., Wang H., Luber B., Alani R.M. (2014). Detection of circulating tumor DNA in early- and late-stage human malignancies. Sci. Trans. Med..

[101] Reinert T., Henriksen T.V., Christensen E., Sharma S., Salari R., Sethi H., Knudsen M., Nordentoft I., Wu H.T., Tin A.S. (2019). Analysis of plasma cell-free DNA by ultradeep sequencing in patients with stages I to III colorectal cancer. JAMA Oncol..

[102] Tie J., Cohen J.D., Wang Y., Li L., Christie M., Simons K., Elsaleh H., Kosmider S., Wong R., Yip D. (2019). Serial circulating tumour DNA analysis during multimodality treatment of locally advanced rectal cancer: A prospective biomarker study. Gut.

[103] Tie J., Wang Y., Tomasetti C., Li L., Springer S., Kinde I., Silliman N., Tacey M., Wong H.L., Christie M. (2016). Circulating tumor DNA analysis detects minimal residual disease and predicts recurrence in patients with stage II colon cancer. Sci. Trans. Med..

